# An eighteen-year longitudinal examination of school victimization and weapon use in California secondary schools

**DOI:** 10.1007/s12519-023-00714-w

**Published:** 2023-03-28

**Authors:** Rami Benbenishty, Ron Avi Astor, Ilan Roziner

**Affiliations:** 1 Hebrew University of Jerusalem, Jerusalem, Israel and Universidad Andrés Bello, Santiago, Chile; 2 Crump Chair in Social Welfare, Luskin School of Public Affairs and School of Education and Information Studies, University of California, Los Angeles, Los Angeles, CA 90095 USA; 3 Sackler School of Medicine, Department of Communication Disorders, Tel Aviv University, Tel Aviv, Israel

**Keywords:** School violence, School climate, Weapons

## Abstract

**Background:**

School safety has been a major public health issue in the United States and internationally for more than three decades. Many policies and programs have been developed and implemented to prevent school violence, improve the school climate, and increase safety. There are only a few peer-reviewed studies of changes in school violence over time. The study examined changes over time in school victimization, weapon involvement and school climate, comparing change trajectories by gender and race and different change trajectories among schools.

**Methods:**

A longitudinal study of the biennial California Healthy Kids Survey in secondary schools from 2001 to 2019. The representative sample included 6,219,166 students in grades 7, 9, and 11 (48.8% male) from 3253 schools (66% high schools).

**Results:**

All victimization and weapon involvement items had significant and substantial linear reductions. The largest reduction involved being in a physical fight (from 25.4% to 11.0%). There were reductions in weapon involvement (*d* = 0.46) and victimization (*d* = 0.38). Biased-based victimization only declined slightly (*d* = −0.05). School belongingness and safety increased (*d* = 0.27), adult support increased a small amount (*d* = 0.05), and student participation declined (*d* = −0.10). Changes were smallest among White students. Ninety-five percent of the schools showed the same pattern of reductions.

**Conclusions:**

The findings are in contrast to the public’s concerns that school violence is a growing problem. Reductions in school violence may result from social investment in school safety. A distinction should be made between school shootings and other forms of school violence.

## Introduction

School safety has been a major public health issue in the United States and internationally for more than three decades [1, 2]. The current widely accepted definition of school violence is “any behavior intended to harm, physically or emotionally, individuals in school, their property, or their school's property” [3–5]. This includes face-to-face and electronic media-related victimization, verbal and social bullying, physical violence, stealing, damage to property, expressions of hate, weapon use, sexual harassment, and assault. Indeed, research shows that many students worldwide are regularly exposed to wide-ranging victimization in schools, such as verbal, social (in person and online), physical, and sexual victimization [6, 7]. Some students are targets of bullying because of their looks, ethnicity, race, nationality, sexual orientation, or other biases [8, 9]. Moreover, some students experience weapons on school grounds, such as carrying a weapon such as a gun or a knife, being threatened or injured by a weapon, or seeing another student carrying a weapon at school [10–12].

Victimization at school affects students’ school connectedness, nonattendance, and dropout [3]. Victimization contributes negatively to overall mental health, depression, suicidal behaviors, and subsequent involvement in risky behaviors, such as substance use [13–16].

There is a strong media and public interest in mass shootings in schools. Each school shooting is a devastating act that terrorizes the nation. The national media report these events intensely and frequently [17, 18]. With the recent increase in school shootings [19], there is a growing sense in the public that little has changed in two decades to make schools safe [20–22]. Given the horror of school shootings, there have been few empirical discussions related to increases or decreases in other harmful types of school victimization in the past two decades [23].

During the past two decades, billions of dollars, resources, policies, programs, and community efforts have been focused on reducing victimization and increasing the safe climate in schools worldwide [24, 25]. This represents a wide array of different policies for school violence, including but not limited to zero tolerance, prevention-oriented social-emotional programs, restorative justice approaches and trauma-informing school strategies [4]. There is a great need for research examining school violence time trends after these types of policies and approaches have been implemented at the population level [3].

To inform public health school safety policies and decisionmaking, this study examined a large sample of secondary schools and students during the past two decades in California. Specifically, the study examined whether there are consistent trends in the prevalence of specific types of victimization and school climate and whether these trends differ by gender and ethnic affiliation.

Several national surveillance systems track different types of school violence and crime [26, 27]. An annual report on indicators of school crime and safety compiles reports from several resources and provides detailed information on the prevalence of 22 relevant indicators through the years (some indicators starting as early as 1992) [26]. Based on this report, nationally, there have been consistent reductions over time in most indicators of victimization on school grounds. From 1992 to 2019, the total victimization rate and rates of specific crimes—thefts and violent victimizations—declined for students aged 12–18 years from 18.1% in 1992 to 3.0% in 2019, more than an 80% decrease [24]. Having been in a physical fight in school decreased from 11.09% in 2009 to 8.03% in 2019, and carrying a weapon on school property during the previous 30 days declined from 5.6% to 2.8% [26].

These US annual surveillance reports are important in showing clear national trends. Nonetheless, they are limited. First, they represent the nation as a whole and thus are less useful in understanding regional variations among states. For instance, estimates regarding being involved in a physical fight in school in 2019 ranged from 27.3% in Mississippi to 16.7% in Hawaii [26, 27]. Furthermore, complementary Youth Risk Behavior Surveillance System state-level estimates available for 34 states are based on relatively small samples, making estimates far less reliable. For instance, the California sample included only 1295 students and the 95% confidence interval for having been involved in a physical fight in school was 6.32 to 30.65, an extremely wide range [28]. Only a handful of studies have examined state-level trends in school victimization, and they have been limited in sample size and the length of time they cover [23].

National or state-level data showing consistent trends of reductions in the prevalence of school victimization are mainly based on student-level data and do not inform policymakers whether, among these national and state trends, some schools show different patterns. For example, an empirical understanding of whether there are schools or districts in which violence is increasing rather than decreasing is lacking. This information is essential for state-level policymakers who need to prioritize districts and schools and allocate necessary resources [29, 30].

Another important limitation is that many national surveillance surveys, such as those reporting indicators of school crime and safety, do not cover relevant aspects of school climate. Although there are multiple conceptualizations and measures of school climate [30, 31], there is an agreement in the research literature that students’ sense of safety, school belongingness, perceived support from adults in school, and the degree to which they have opportunities to participate and help make decisions and choices are central climate dimensions that could reduce school violence [32, 33]. These climate dimensions make schools feel more welcoming, caring, and safe [34]. Promoting school climate is part of the World Health Organization’s health-promoting schools framework [35]. School climate could be considered a proximal determinant of risks associated with health and exposure to violence in school and should be part of the surveillance system. Trends showing declines in victimization should correspond to positive trends in school climate variables. Do declines in victimization and increases in school climate occur across all students? Are these trends similar across schools? These issues have yet to be studied using large-scale surveillance data. Social-ecological theoretical frameworks have long called for studies that examine changes in long-term trends of school violence as a way to improve theory, research and policy. The same theoretical frameworks suggest more studies exploring shifts in school violence trends with samples that represent large regions, such as states. There are fewer than a handful of empirical studies in the peer-reviewed literature that have examined these issues.

The current study addressed these gaps in knowledge by analyzing a very large sample (close to a population-level sample) of schools and students in California for more than 18 years to examine (1) changes over time in students’ reports of school victimization, weapon involvement, and school climate; (2) different trajectories among boys and girls and among students from different ethnicities and cultural groups, and (3) how many schools had similar or different patterns of changes over time.

## Methods

### Procedure and sample

The data used in this study are from the California Healthy Kids Survey, a modular survey instrument developed by WestEd in collaboration with the California Department of Education and used biannually since 2001. The survey is conducted as a census among all school districts, schools, and students in the relevant grades. Every school year (e.g., 2001–2002), a survey is carried out in a group of districts and in the following year among the rest of the districts. Two consecutive school years (i.e., 2001–2002 and 2002–2003) create a representative dataset that includes most school districts and schools in all counties across the state. Student participation is voluntary, anonymous, and confidential [36, 37]. Prior statewide studies report that approximately 85% of school districts in California participate in data collection [38, 39]. Multiple studies using CHKS data from a Consortium of several school districts in the Southern California region report an 87% student-level response rate [40–42].

The authors merged all data from secondary schools (middle and high schools) from the period 2001–2002 to the period 2018–2019 school years (data are presented for each two consecutive school years that form a representative sample). The total sample includes 6,219,166 students (48.8% male) from 3253 schools—66% from high schools and the rest from middle schools.

### Measures

#### Victimization

Students responded to questions regarding their victimization at school in the past 12 months using a four-point scale: 1 = 0 times, 2 = 1 time, 3 = 2 or 3 times, and 4 = 4 or more.

##### Verbal, social, and physical victimization

Examples of this type of victimization include being pushed, shoved, slapped, hit, or kicked at school; being afraid of being beaten up at school; and having mean rumors or lies spread at school and through the internet (*α* = 0.78).

##### Discrimination-based harassment or bullying

Students were asked whether they were harassed or bullied on school property for six reasons: race, religion, gender, gender identity, disability, and other (*α* = 0.73).

##### Weapon involvement

Four questions were asked regarding weapons: in the last 12 months, how many times a. the student carried a gun in school; b. carried another weapon; c. was threatened or injured with a weapon; and d. saw other students carrying a gun on school grounds (*α* = 0.71).

#### School climate

##### Feeling safe at school

Students were asked to what extent they agree with the statement, “I feel safe in my school” (1 = strongly disagree to 5 = strongly agree).

##### Adult support

This variable was computed as a mean of six items asking about aspects of adult support (e.g., “At my school … there is a teacher or adult who truly cares about me,” “who tells me when I do a good job,” and “who notices when I’m not there”). Responses were provided on a scale from 1 (not at all true) to 4 (very much true; *α* = 0.89).

##### School belongingness

This index, computed as a mean of five questions, assessed students’ feelings toward their school (e.g., “I feel close to the people at this school” and “I am happy to be at this school”) using a 5-point scale (1 = strongly disagree to 5 = strongly agree; *α* = 0.79).

##### Participation

This index, computed as a mean of three items, described student participation in school (e.g., “At school I help decide things” and “I do things at school that make a difference”) based on a 4-point scale (1 = not at all true to 4 = very much true; *α* = 0.76).

### Statistical analyses

Descriptive statistics were computed for all types and indexes of victimization and climate. Time trends were assessed using three complementary methods: (1) Change between the first and last year of the survey was computed as the difference between the last and initial frequencies as a proportion of the initial frequency (last-first/(first)); (2) Cohen’s *d* was computed as a measure of the effect size of last–first. For differences between percentages, we used *d* = 2*sqrt(arcsin(last%))-2*sqrt(arcsin(first%)), whereas for differences between means, we used *d* = ((mean(last)-mean(first))/(pooled SD), and (3) For continuous variables, regression analyses were conducted to assess linear trends over time considering all 18 data points, yielding *B*, standard error (SE), and *β* coefficients of the time trends. These analyses were carried out with SAS PROC SURVEYREG, controlling for school level (middle or high school), gender, and ethnicity, considering the clustered design of the sample (students nested in schools). Separate analyses were conducted to assess the interaction of time with gender and race/ethnicity. Effect sizes (Cohen’s *d*) and time trends (*B* and *β* coefficients) were computed separately for boys, girls, and each ethnicity, along with regression interaction effects for gender and ethnicity (boys and White were the reference groups).

To identify groups of schools with potentially different trajectories of change across time, we estimated unconditional latent class growth models for each dependent variable using Mplus version 8.6 software with the MLR estimator, fitting models with one to three latent classes. In each growth model, the intercept, linear slope, and quadratic slope of change over time were tested [43–45].

The study was reviewed by the institutional review board (IRB) of the second author’s university and was exempted because it is a secondary analysis of publicly available data.

### Findings

Table 1 presents the percentage of students reporting each type of victimization at each biannual point in time, measures of change over time (proportion of change and Cohen’s *d*), and regression coefficients of the time trend. (Similar tables were generated for each race and gender separately, but due to space limitations, they are not presented here and are available upon request).

**Table 1:** Victimization types over time (N, %) by school year, change score (%), and linear time effects

Questions	2001–2003	2003–2005	2005–2007	2007–2009	2009–2011	2011–2013	2013–2015	2015–2017	2017–2019	Measures of change over time			
	(*N* = 320,799), %	(*N* = 553,025), %	(*N* = 616,113), %	(*N* = 725,410), %	(*N* = 689,108), %	(*N* = 547,336), %	(*N* = 822,016), %	(*N* = 919,484), %	(*N* = 1,025,875), %	First to last year effects		Regression coefficients	
										Δ%	*d*	*B*	*β*
Been in a physical fight at school?^a^	25.4	23.7	24.1	22.3	20.0	17.1	13.2	10.8	11.0	–56.2	–0.38	–0.021	–0.143
Been pushed, shoved, slapped, hit, or kicked at school?^a^	37.9	36.5	33.9	33.6	30.6	28,0	23.1	21.5	20.9	–44.3	–0.38	–0.023	–0.124
Had mean rumors or lies spread about you at school?^a^	45.5	44.2	43.6	43.0	39.6	37.5	35.3	34.2	34.0	–24.4	–0.24	–0.012	–0.059
Had sexual jokes, comments, or gestures made to you at school?^a^	50.9	47.3	45.5	45.4	43.7	38.2	33.3	30.1	28.1	–43.0	–0.45	–0.032	–0.141
Been made fun of because of your looks or the way you talk at school?^a^	41.0	39.6	37.9	38.8	37.6	36.2	33.6	30.9	30.3	–25.0	–0.22	–0.012	–0.055
Had your property stolen or damaged, at school?^a^	17.6	16.8	15.6	14.9	13.0	10.3	7.6	5.3	5.0	–43.0	–0.31	–0.014	–0.094
Carrying a gun on school property?^a^	4.3	4.2	5.0	5.2	4.9	4.1	2.8	1.6	1.3	–69.8	–0.19	–0.005	–0.067
Carrying a weapon such as a knife on school property?^a^	11.9	11.8	12.1	10.1	9.7	8.1	6.4	4.5	3.8	–67.8	–0.31	–0.012	–0.106
Been threatened or injured with a weapon at school?^a^	9.1	8.5	9.3	8.6	8.3	6.9	5.3	3.8	3.7	–58.9	–0.23	–0.007	–0.078
Seen someone carrying a weapon at school?^a^	31.6	35.0	33.7	29.2	27.3	23.7	17.7	13.6	12.1	–61.2	–0.48	–0.032	–0.193
Been afraid of being beaten up at school?^b^	23.5	22.6	21.4	21.5	19.5	18.1	14.8	13.5	14.3	–38.6	–0.24	–0.009	–0.065

All percentages are computed for “at least once” during the last year. Coefficients are computed using the original scales. All SE(*B*) ≤ 0.001; all *β* significant at *P* < 0.001. Given space limitations and that the sample is very large and confidence intervals are very small, only point estimates are provided. Confidence intervals for all coefficients are available upon request

^a^Scale: 1 = 0 times, 2 = 1 time, 3 = 2–3 times, 4 = more than 3 times

^b^Scale: 1 = strongly disagree to 5 = strongly agree

All victimization and weapon involvement items declined between 2001–2003 and 2017–2019. The largest reduction was for being involved in a physical fight (from 25.4% in 2001–2003 to 11.0% in 2017–2019, a reduction of 14.4 percentage points or approximately 56%, *d* = −0.38). Weapon-related behaviors also dropped sharply—e.g., carrying a gun on school grounds dropped from 4.3% to 1.3%, approximately 70% (*d* = −0.19). Similar reductions were evident for carrying another kind of weapon (67.8%, *d* = −0.31), seeing someone with a weapon on school grounds (61.2%, *d* = −0.48), and being threatened or injured with a weapon (58.9%. *d* = −0.28). Smaller reductions were noted for some nonphysical types of victimization, such as being made fun of because of looks (25.0%, *d* = −0.22) and having mean rumors spread (24.4%, *d* = −0.24). Effect sizes were small to medium—for instance, for seeing someone carrying a weapon on school grounds (*d* = −0.48), carrying a weapon (not a gun) on school grounds (*d* = −0.31), and being in a physical fight (*d* = −0.48). The smallest reduction was in the index of discrimination-based victimization (*d* = −0.017).

Table 2 presents the means and standard deviations of victimization and climate indexes, Cohen’s *d*, and unstandardized and standardized regression coefficients. Regression analyses revealed significant time effects for most of the indexes. The only significant effects were linear. The unstandardized coefficients were −0.017 for victimization, −0.014 for weapon involvement, and 0.001 for bias-based victimization. Note that coefficients indicate per-year change; hence, they should be multiplied by the 18-year span to assess the full effect of the reductions over time.

**Table 2:** Indexes of victimization and climate (*M*, SD) by school year, Cohen’s *d*, and linear time effects (*B*, β)

Variables	2001 –2003	2003–2005	2005–2007	2007–2009	2009–2011	2011–2013	2013–2015	2015–2017	2017–2019	Measures of change over time		
	(*N* = 320,799)	(*N* = 553,025)	(*N* = 616,113)	(*N* = 725,410)	(*N* = 689,108)	(*N* = 547,336)	(*N* = 822,016)	(*N* = 919,484)	(*N* = 1,025,875)	First to last	Regression coefficients	
	*M*, SD	*M*, SD	*M*, SD	*M*, SD	*M*, SD	*M*, SD	*M*, SD	*M*, SD	*M*, SD	*d*	*B*	*β*
Victimization^a^	1.68	1.68	1.65	1.68	1.63	1.58	1.50	1.46	1.44	–0.38	–0.017	–0.128
	0.68	0.66	0.66	0.71	0.70	0.69	0.65	0.62	0.62			
Weapon involvement^a^	1.24	1.27	1.27	1.25	1.23	1.20	1.14	1.10	1.09	–0.46	–0.014	–0.161
	0.48	0.49	0.51	0.51	0.51	0.47	0.39	0.32	0.29			
Discrimination victim^a^	1.16	1.17	1.18	1.20	1.20	1.18	1.17	1.16	1.15	–0.05	–0.001	–0.017
	0.37	0.38	0.40	0.46	0.45	0.44	0.41	0.38	0.37			
Adult Support^b^	2.90	2.83	2.84	2.96	2.95	2.97	2.89	2.91	2.87	0.05	0.003	0.016
	0.85	0.82	0.83	0.75	0.75	0.75	0.78	0.78	0.78			
Belongingness^b^	–	3.33	3.37	3.50	3.53	3.56	3.55	3.62	3.56	0.27	0.018	0.098
		0.88	0.90	0.84	0.84	0.84	0.83	0.85	0.84			
Participation^b^	2.29	2.24	2.26	2.25	2.25	2.27	2.24	2.24	2.16	–0.10	–0.003	–0.019
	0.86	0.86	0.87	0.83	0.83	0.83	0.84	0.85	0.81			
Feeling safe at school^c^	–	3.34	3.37	3.50	3.53	3.60	3.67	3.72	3.62	0.27	0.025	0.110
		1.04	1.06	1.13	1.12	1.10	1.04	1.04	1.04			

All *SE* (*B*) ≤ 0.001; all *β* significant at *P* < 0.001. Given space limitations and that the sample is very large and confidence intervals are very small, only point estimates are provided. Confidence intervals for all coefficients are available upon request. *M* mean

^a^Scale regarding past 12 months: 1 = 0 times, 2 = 1 time, 3 = 2–3 times, 4 = 4 times or more

^b^Scale: 1 = not at all true, 2 = a little true, 3 = pretty much true, 4 = very much true

^c^Scale: 1 = strongly disagree to 5 = strongly agree

The indexes capturing victimization and weapon involvement in Table 2 show sizeable time effects. For instance, Cohen’s *d* was 0.46 for the index of weapon involvement and 0.38 for victimization. There were also significant increases in students’ sense of belonging to the school and feeling safe at school (both *d* = 0.27). In contrast, adult support showed only a small increase over time (*d* = 0.05), and participation in school slightly declined over time (*d* = −0.10).

To examine whether there are groups of students whose change over time was different than others, we computed the interactions of time with gender and race (Table 3). All gender interaction terms were significant, indicating a consistent pattern of boys changing over time more than girls. The largest difference between boys and girls was for reductions in weapon-related behaviors—e.g., carrying weapons other than guns (boys: *d* = −0.44, girls: *d* = −0.22).

**Table 3:** Time main effects and interactions of school victimization and climate by gender and ethnicity (*B*, *β*, and Cohen’s *d*)

Variables		Gender			Ethnicity												
		Boys	Girls		White	Native American		Hawaiian or Pacific Islander		African American		Asian		Hispanic		Others	
		Main	Main	Int	Main	Main	Int	Main	Int	Main	Int	Main	Int	Main	Int	Main	Int
Been in a physical fight at school?^a^	*B*	–0.014	–0.028	–0.015	–0.015	–0.027	–0.009	–0.025	–0.010	–0.027	–0.011	–0.016	**0.001**	–0.025	–0.009	–0.019	–0.002
	*β*	–0.117	–0.170	–0.052	–0.126	–0.149	–0.007	–0.157	–0.008	–0.143	–0.015	–0.140	**–** **0.001**	–0.159	–0.033	–0.126	–0.005
	*d*	–0.49	–0.28		–0.35	–0.43		–0.48		–0.39		–0.42		–0.42		–0.36	
Been pushed, shoved, slapped, hit or at school?^a^	*B*	–0.016	–0.031	–0.016	–0.023	–0.028	**–** **0.001**	–0.023	**0.001**	–0.019	0.005	–0.022	0.002	–0.023	**0.001**	–0.024	**0.001**
	*β*	–0.095	–0.152	–0.044	–0.130	–0.129	**–** **0.001**	–0.117	**0.001**	–0.095	0.005	–0.129	0.003	–0.121	**0.001**	–0.125	**0.001**
	*d*	–0.49	–0.28		–0.21	–0.23		–0.23		–0.22		–0.24		–0.27		–0.25	
Had mean rumors or lies spread about you at school?^a^	*B*	–0.010	–0.014	–0.005	–0.010	–0.012	**–** **0.002**	–0.014	–0.004	–0.013	–0.003	–0.013	–0.004	–0.013	–0.004	–0.009	**0.001**
	*β*	–0.046	–0.075	–0.012	–0.048	–0.056	**–0.001**	–0.063	–0.002	–0.062	–0.003	–0.071	–0.006	–0.065	–0.010	–0.045	**0.001**
	*d*	–0.27	–0.19		–0.22	–0.20		–0.21		–0.26		–0.26		–0.24		–0.18	
Had sexual jokes, comments, or gestures made to you at school?^a^	*B*	–0.034	–0.030	0.003	–0.033	–0.028	**0.002**	–0.033	**0.001**	–0.039	–0.007	–0.026	0.006	–0.034	–0.002	–0.028	0.003
	*β*	–0.146	–0.136	0.007	–0.143	–0.119	**0.001**	–0.134	**0.001**	–0.162	–0.006	–0.122	0.009	–0.147	–0.005	–0.119	0.004
	*d*	–0.46	–0.44		–0.48	–0.41		–0.42		–0.50		–0.40		–0.45		–0.32	
Been made fun of because of your looks or the way you talk at school?^a^	*B*	–0.007	–0.017	–0.010	–0.011	–0.009	**0.003**	–0.006	0.005	–0.011	**0.001**	–0.015	–0.004	–0.012	–0.001	–0.011	**0.001**
	*β*	–0.032	–0.079	–0.024	–0.051	–0.042	**0.001**	–0.027	0.003	–0.047	**0.001**	–0.072	–0.006	–0.055	–0.003	–0.052	**0.001**
	*d*	–0.28	–0.17		–0.22	–0.14		–0.13		–0.21		–0.24		–0.22		–0.19	
Had your property stolen or deliberately damaged at school?^a^	*B*	–0.010	–0.018	–0.007	–0.014	–0.015	**0.001**	–0.016	**–0.002**	–0.014	**0.000**	–0.017	–0.003	–0.013	**0.001**	–0.013	**0.001**
	*β*	–0.076	–0.111	–0.025	–0.100	–0.087	**0.000**	–0.096	**–0.002**	–0.088	**0.000**	–0.112	–0.005	–0.088	**0.003**	–0.087	**0.001**
	*d*	–0.37	–0.27		–0.34	–0.29		–0.33		–0.28		–0.36		–0.27		–0.26	
Carrying a gun on school property?^a^	*B*	–0.002	–0.008	–0.006	–0.003	–0.009	–0.005	–0.007	–0.004	–0.009	0.000	–0.004	–0.005	–0.006	–0.002	–0.005	–0.002
	*β*	–0.043	–0.087	–0.038	–0.049	–0.080	–0.007	–0.076	–0.006	–0.080	–0.002	–0.062	–0.013	–0.077	–0.007	–0.064	–0.007
	*d*	–0.24	–0.14		–0.15	–0.30		–0.26		–0.21		–0.21		–0.23		–0.20	
Carrying a weapon such as knife or club school property?^a^	*B*	–0.006	–0.018	–0.012	–0.009	–0.016	–0.006	–0.014	–0.006	–0.016	–0.007	–0.008	0.000	–0.015	–0.006	–0.011	–0.003
	*β*	–0.073	–0.135	–0.053	–0.084	–0.112	–0.006	–0.113	–0.006	–0.118	–0.012	–0.094	0.001	–0.121	–0.027	–0.098	–0.008
	*d*	–0.40	–0.22		–0.27	–0.38		–0.38		–0.35		–0.33		–0.36		–0.29	
Been threatened or injured with a weapon at school?^a^	*B*	–0.004	–0.010	–0.006	–0.005	–0.011	–0.005	–0.009	–0.004	–0.010	–0.005	–0.005	**0.000**	–0.008	–0.003	–0.007	–0.002
	*β*	–0.054	–0.098	–0.036	–0.064	–0.088	–0.006	–0.083	–0.005	–0.089	–0.011	–0.075	**–0.001**	–0.085	–0.016	–0.075	–0.007
	*d*	–0.28	–0.17		–0.21	–0.30		–0.27		–0.24		–0.26		–0.23		–0.23	
Seen someone carrying a weapon at school?^a^	*B*	–0.025	–0.038	–0.013	–0.024	–0.031	–0.007	–0.035	–0.011	–0.039	–0.015	–0.024	**–0.001**	–0.038	–0.015	–0.028	–0.005
	*β*	–0.174	–0.213	–0.040	–0.158	–0.171	–0.005	–0.196	–0.009	–0.210	–0.018	–0.177	**–0.001**	–0.219	–0.047	–0.170	–0.009
	*d*	–0.50	–0.43		–0.43	–0.52		–0.54		–0.56		–0.52		–0.56		–0.42	
Been afraid of being beaten up at school?^a^	*B*	–0.007	–0.011	–0.004	–0.008	–0.010	**–0.001**	–0.010	–0.002	–0.008	0.000	–0.009	–0.001	–0.010	–0.002	–0.009	–0.001
	*β*	–0.053	–0.077	–0.015	–0.057	–0.058	**–0.001**	–0.064	–0.002	–0.057	0.000	–0.066	–0.003	–0.072	–0.009	–0.061	–0.003
	*d*	–0.27	–0.21		–0.21	–0.23		–0.23		–0.22		–0.24		–0.27		–0.25	
Moderate victim^a^	*B*	–0.014	–0.020	–0.007	–0.016	–0.017	**0.000**	–0.017	**–0.001**	–0.017	**–0.001**	–0.017	–0.001	–0.017	–0.002	–0.015	**0.000**
	β	–0.107	–0.148	–0.027	–0.125	–0.112	**0.000**	–0.121	**–0.001**	–0.124	**–0.001**	–0.136	–0.003	–0.131	–0.007	–0.114	**0.001**
	*d*	–0.44	–0.30		–0.38	–0.37		–0.35		–0.36		–0.41		–0.35		–0.29	
Weapon involved^a^	*B*	–0.009	–0.019	–0.009	–0.010	–0.017	–0.006	–0.017	–0.006	–0.018	–0.008	–0.010	**0.000**	–0.017	–0.007	–0.013	–0.003
	*β*	–0.140	–0.184	–0.055	–0.133	–0.150	–0.007	–0.165	–0.009	–0.169	–0.018	–0.152	**–0.001**	–0.183	–0.041	–0.146	–0.010
	*d*	–0.47	–0.36		–0.36	–0.55		–0.48		–0.43		–0.47		–0.46		–0.37	
Discrimination victim^a^	*B*	0.001	–0.004	–0.005	0.000	–0.002	–0.002	–0.001	**–0.001**	–0.001	**–0.001**	–0.002	–0.002	–0.002	–0.002	–0.001	–0.001
	β	0.012	–0.043	–0.029	0.001	–0.021	–0.002	–0.011	**–0.002**	–0.007	**–0.002**	–0.020	–0.007	–0.027	–0.014	–0.014	–0.005
	*d*	–0.10	0.03		–0.02	–0.06		–0.04		0.01		–0.06		–0.04		–0.01	
Adult support^b^	*B*	–0.002	0.007	0.009	0.003	0.004	**–0.001**	0.005	**0.002**	0.004	**0.001**	0.005	**0.001**	0.002	–0.002	0.001	–0.002
	*β*	–0.011	0.045	0.029	0.023	0.026	**–0.001**	0.033	**0.001**	0.025	**0.001**	0.030	**0.002**	0.009	–0.006	0.010	–0.005
	*d*	0.07	–0.12		–0.01	0.03		0.04		–0.03		0.05		–0.02		–0.05	
Belongingness^b^	*B*	0.012	0.024	0.013	0.017	0.024	0.005	0.019	**0.002**	0.023	0.003	0.021	0.005	0.017	**0.000**	0.017	**–0.001**
	*β*	0.068	0.129	0.036	0.098	0.116	0.003	0.104	**0.001**	0.113	0.006	0.121	0.005	0.092	**–0.001**	0.096	**–0.002**
	*d*	0.36	0.19		0.27	0.34		0.30		0.32		0.37		0.27		0.27	
Participation^b^	*B*	–0.004	–0.002	0.003	–0.003	–0.004	**–0.001**	–0.005	**–0.001**	–0.003	**0.001**	0.000	0.004	–0.003	**0.000**	–0.006	–0.003
	*β*	–0.026	–0.013	0.009	–0.020	–0.022	**–0.001**	–0.026	**–0.001**	–0.018	**0.001**	0.000	0.007	–0.019	**0.001**	–0.037	–0.005
	*d*	–0.08	–0.11		–0.15	–0.12		–0.13		–0.12		–0.04		–0.14		–0.18	
Feeling safe at school^c^	*B*	0.020	0.031	0.011	0.023	0.032	0.007	0.025	**0.002**	0.032	0.009	0.026	0.002	0.026	0.003	0.024	**0.000**
	*β*	0.092	0.128	0.024	0.106	0.120	0.003	0.105	**0.001**	0.125	0.007	0.117	0.003	0.112	0.007	0.104	**0.000**
	*d*	0.32	0.21		0.24	0.33		0.27		0.33		0.33		0.29		0.26	

All SE (*B*) ≤ 0.001; all *β* significant at *P* < 0.001 except for *β* in bold, which are not significant. Boy was the reference category for gender and White was the reference category for ethnicity. Given space limitations and that the sample is very large and confidence intervals are very small, only point estimates are provided. Confidence intervals for all coefficients are available upon request. *Int* interactions

^a^Scale: regarding past 12 months: 1 = 0 times, 2 = 1 time, 3 = 2–3 times, 4 = 4 times or more

^b^Scale: 1 = not at all true, 2 = a little true, 3 = pretty much true, 4 = very much true

^c^Scale: 1 = strongly disagree to 5 = strongly agree

The interactions of time with race and ethnicity revealed more complex patterns. Overall, and quite consistently, the effect sizes of change over time were the smallest among White students (except when compared to students in the “other” race category). Additionally, African American and Hispanic students tended to have more reductions in victimization and weapon-related behaviors and more improvements in school climate than White students.

The findings of latent class growth models estimated for all indexes were consistent: at least 95% of the schools showed a linear trend of reductions in victimization and weapon-related behaviors, increases in feeling safe in school, school belongingness, and stable adult support; and a small reduction in student participation. Given that there were almost no variations in school-level latent class growth models, we did not explore differences in models between school types (e.g., between urban and nonurban schools).

## Discussion

California secondary schools had massive reductions in all forms of verbal, psychological, property, physical, and weapons involvement behaviors during the 18-year period examined. This was especially strong in the physical victimization and weapons use areas (and much less in bias-based victimization). The consistent reductions were evident in more than 95% of California schools, affecting almost all schools and regions in California, and not in wealthy suburban schools only. Both boys and girls showed strong reductions, with boys showing stronger decreases. Extensive reductions were evident in all cultural, racial, and ethnic groups. In fact, the reductions in victimization among all other ethnic groups were greater than those among White students (except for those indicating “other” ethnicity), especially regarding reduced involvement with weapons. More research is needed to better understand the differential reductions for the various ethnic groups. However, given how ethnically homogenous California schools are, it is possible that more systemic efforts were placed in urban, high need, and schools with high proportions of Latinx and Black students. However, this possibility needs to be better explored by future research.

This consistent set of findings, based on a very large and representative 18-year sample, goes in the opposite direction of the public’s concern and perception that school violence was a growing problem during these two decades [46–48]. Given the massive reductions in victimization overall, it is quite likely that the impressions of the public and policymakers regarding school safety and the effectiveness of state and national investments are associated with the escalation of school shootings and sustained and intense media coverage of mass shootings, rather than other forms of school victimization. The reductions in school violence raise the possibility that the efforts, norm shifts, and two decades of massive social investment in school safety contributed to dramatically less victimization for California’s students. The sharp declines in rates of victimization at school should be part of the public policy discourse that is currently overshadowed by school shootings. California’s policies have made billions of dollars of investment in school safety issues available. These have likely increased awareness and capacity, changed behavior practices, and provided evidence-based ways to address the problem. It is possible that these collective policies over the past two decades have contributed to the reductions during the same period. However, more detailed and nuanced mixed methods and qualitative studies are needed to better understand whether the implementation of these collective policies possibly reduced victimization levels. Furthermore, it is important to study to what extent findings in California are similar to other regions that may implement different programs and policies. If the efforts in California made a difference, it is important for policymakers and the research literature to acknowledge the possible benefits of the efforts invested in violence prevention and sustain them in the future.

We propose, therefore, that a clear distinction should be made between mass school shootings and other forms of school violence. A conceptual, methodological, and empirical distinction between school shootings and other forms of school violence would help identify different psychological, social, and ecological mechanisms that may lead to these potentially separate phenomena [49, 50]. It would also sharpen the policy and practical implications derived from research, given that reductions in forms of school violence not involving shootings are strong and consistent.

Biased-based victimization has not changed as much as all other types of victimization. It encompasses a wide set of biases, including gender and gender identity, race, religion, and disability. This finding may reflect the societal struggle with divisive policies and disagreements on basic values that affect the school environment [51–53]. It should prompt a review of current interventions to develop ways in which education could lead to fewer bias-driven types of victimization.

It is important to note that as could be expected based on the theoretical literature, reductions in school violence were accompanied by an improving sense of safety and school belongingness over time. In contrast, other aspects of a positive school climate, such as adult support and student participation, did not improve over time. This finding, based on a large and longitudinal dataset, requires further investigation to review current claims about the role of adult support and student participation in preventing school violence. Perhaps developing school climate interventions that focus more on belonging and a sense of safety would produce stronger reductions in victimization. Adult support and student participation aspects of school climate may have other important educational advantages, but they may not contribute to violence prevention [54]. More research is needed to specifically examine the relationships between the effective components of school climate and the wide array of school safety interventions. Many interventions claim to impact school climate, but there is little empirical evidence examining the reciprocal relationships between school climate and evidence-based interventions.

In conclusion, this study covered the period before the COVID-19 pandemic. There are several indications that the pandemic led to multiple negative mental health outcomes for children and adolescents and that returning to school may be associated with higher levels of school violence [55–58]. This potential increase in school violence should be monitored closely. Schools may continue to need more resources to address the increasing burden of COVID-19 mental health issues. It is important to learn from the policies and interventions that have helped reduce school violence in the last two decades to face these new challenges [59].

## Data Availability

Data files will be provided in response to any reasonable request.
